# Enhancing Recruitment in Urinary Incontinence Care: Insights from the EvidenceNOW Managing Urinary Incontinence Initiative

**DOI:** 10.1007/s00192-026-06658-9

**Published:** 2026-04-30

**Authors:** Rachel M. Burns, Nipher Malika, Shona Olalere Oluwatola, Gabriela Alvarado, Martha Timmer, Sangeeta Ahluwalia

**Affiliations:** 1 RAND Corporation, 4570 5Th Ave, Pittsburgh, PA 15213 USA; 2 RAND Corporation, 1776 Main St., Santa Monica, CA 90401 USA; 3 RAND Corporation, 1200 S Hayes St., Arlington, VA 22202 USA

**Keywords:** Evidence-based practice, Primary care, Recruitment strategies, Screening, Urinary incontinence, Women’s health

## Abstract

**Introduction and Hypothesis:**

Urinary incontinence (UI) affects over half of adult women but is widely underdiagnosed and undertreated in primary care due to stigma, normalization, and limited routine screening. Identifying effective recruitment strategies is essential to improving UI care in these settings.

**Methods:**

We conducted semi-structured interviews and focus groups with grantee leaders and practice facilitators (*n* = 32), site visits and virtual interviews with providers and staff (*n* = 47) and collected quarterly quantitative recruitment and retention data from five grantees participating in a nationwide UI initiative. Qualitative data were analyzed thematically using the Consolidated Framework for Implementation Research. Quantitative data, including 1950 enrolled practices and 134,852 screened patients, were summarized with descriptive statistics.

**Results:**

No single recruitment strategy was universally effective. All grantees utilized approaches such as leveraging professional relationships, engaging practice facilitators, and conducting in-person meetings, which were consistently linked to higher practice and provider recruitment. Leadership engagement facilitated recruitment success, while use of incentives and broad outreach alone were insufficient. Patient recruitment was most successful when UI screening was embedded in routine intake workflows. Across all grantees, 21% of screened patients tested positive for UI. Recruitment challenges included approval delays in large systems and increased workload related to integrating screening into routine care.

**Conclusions:**

Successful UI intervention recruitment in primary care requires combining leadership support, workflow integration, and tailored approaches. Limitations include reliance on aggregate data and absence of patient-reported perspectives or subgroup analyses. Future research should further investigate strategies to advance equitable UI care delivery.

## Introduction

More than half of adult women experience urinary incontinence (UI), characterized by involuntary loss of bladder control and urinary leakage [1–4]. Despite the availability of safe and effective treatments, considerable gaps remain in comprehensive incidence and prevalence data, primarily due to underreporting. Many women neither seek nor receive care for their UI symptoms [5–7], often because UI is wrongly perceived as a normal part of aging and rarely screened for or openly discussed by clinicians [8–14]. This convergence of stigma, symptom normalization, and lack of routine screening leads to significant unmet clinical needs and missed opportunities for timely intervention in primary care. Compounding these challenges, many primary care practices lack the necessary protocols, training, and resources to effectively identify and manage UI, further exacerbating gaps in care [15]. Routine screening is rarely implemented, and clinicians may not feel equipped to address UI, resulting in the continued marginalization of patients with UI [16]. Overcoming these barriers requires systematic and effective interventions that promote regular UI screening, reduce stigma, and foster open communication between clinicians and patients.

In recognition of these needs, the Agency for Healthcare Research and Quality (AHRQ) launched the EvidenceNOW Managing Urinary Incontinence (MUI) initiative in early 2022. This national initiative supports primary care practices in implementing evidence-based, nonsurgical interventions for UI in adult women, leveraging proven implementation strategies to enhance the delivery of high-quality UI care [17, 18]. However, integrating such interventions in primary care remains challenging due to time constraints, competing demands, and limited resources. Busy practice environments, structural variability, and diverse patient populations further complicate the adoption of standardized care protocols [19, 20]. To address these complexities, five institutions across the United States were selected to implement and disseminate evidence-based practices for UI screening, management, and referral, with an aim to minimize the operational burden on practices and providers [18].

Rigorous evaluation of these efforts is vital for understanding which recruitment and implementation strategies are most successful, identifying persistent barriers, and refining approaches for widespread adoption. Recruiting practices and patients into such initiatives presents its own challenges, including geographic dispersion, heterogeneity in healthcare structures, and varying levels of staff engagement and willingness to participate [21]. In this study, we examine the recruitment strategies deployed for practices, providers, and patients in UI screening, management, and referral interventions across diverse healthcare settings in the United States. Using a mixed-methods approach, we aimed to determine which strategies are most effective in disseminating and implementing evidence-based, nonsurgical UI treatments within primary care.

## Methods

### Study Setting

This evaluation examined the recruitment strategies for five grantee projects funded by AHRQ to disseminate and implement the evidence in primary care practices for nonsurgical treatment of UI in women. Grantee A used automated screening for UI using an online patient portal and shared decision-making assistance for UI treatment at an academic-based, integrated health system. Grantee B used streamlined practice facilitation to increase screening and treatment of UI at multiple health systems across a state in the Midwest. Grantee C employed a four-pronged approach of academic detailing, clinical decision support, electronic referral, and advanced practice provider co-management to increase screening and treatment at multiple health systems. Grantee D focused on nurse navigation and a mobile chatbot (an interactive artificial intelligence-based conversation platform) to address treatment through self-management efforts at an academic-based, integrated health system; and Grantee E which used mobile Health UI treatment application and an online toolkit that provided access to evidence-based nonsurgical UI treatment targeting the Veterans Affairs (VA) healthcare system across several states [18].

### Study Design

We used a mixed-methods approach to comprehensively analyze recruitment strategies and capture both quantitative outcomes and contextual insights [22]. This study received ethical approval from the author’s institutional review board and all participants provided verbal consent prior to interview participation.

#### Qualitative Study Design and Procedures

We conducted semi-structured interviews with leaders and practice facilitators of all five AHRQ grantees, and we conducted site visits and interviews with four grantees (see Table 1).

**Table 1:** Qualitative participants

	*n*
Grantee principal and co-investigator interviews (*n* = 16)	
Primary care clinicians	
Family physician	1
Primary care internist	2
Obstetrician gynecologist	0
Geriatrician	3
Specialists	
Urogynecology	3
Urologist	4
Health care researcher	1
Clinic research manager	2
Practice facilitators (*n* = 16)	
Project/research managers	11
Geriatrician	1
Urologist	1
Urogynecologist	1
Nurse practitioner	1
Researcher	1
Eight site visit interviews* (*n* = 35)	
Office managers	5
Clinicians	9
Nurses	9
Medical assistants	4
Front desk personnel	8
Eight virtual practice interviews* (*n* = 12)	
Office managers	2
Clinicians	9
Front desk personnel	1

*Two site visits and four virtual practice interviews were conducted for each grantee except for one

##### Grantee Leader Interviews

Grantee leaders and co-investigators (*n* = 16) participated in virtual 1-hour interviews, conducted annually over 3 years, to understand their approaches to implementation, identify challenges encountered, and examine strategies that influenced the successful implementation of UI care improvements.

##### Practice Facilitator Interviews

We conducted virtual 1-hour interviews and focus groups with practice facilitators (*n* = 16). These sessions aimed to gather their insights on challenges encountered when working with various types of practices, the resources needed, and the effectiveness of different dissemination and implementation strategies. All interviews and focus groups were audio recorded with permission transcribed verbatim.

##### Site Visits

We conducted in-person site visits with four of the five grantees to discuss barriers and facilitators to implementing evidence-based UI care for women in primary care, as well as practices’ perceptions of the support and resources provided by grantees. However, for one grantee, privacy restrictions within the VA and other limitations prevented us from conducting these site visits. Individual site visits were conducted over the course of 2 days and occurred once per year for 2 years. During these visits, data collection included interviews with providers and staff (*n* = 35), a walkthrough of clinic UI care processes, a review of materials like brochures and screening forms, and a debriefing session within 24–48 h to compile observation notes.

##### Virtual Practice Interviews

The virtual practice interviews (*n* = 12) were conducted with two additional practices per grantee each year to supplement the in-person visits and to broaden the range of practices included in the qualitative sample. Practices were selected to reflect a range of setting characteristics, including number and types of practitioners, urban or rural location, mix of safety net and commercially insured patients, and practice ownership. All practice site visits and interviews were audio-recorded with permission and supplemented by manual notes. A standardized observation template was used to document key interactions and impressions not covered in the interview notes or transcripts. No patient interviews were conducted, and private interactions between patients and staff were neither observed nor recorded. Similarly to site visits, for one grantee, we were unable to conduct interviews due to VA restrictions.

The interviewers were trained in qualitative methods and possessed extensive prior experience interviewing and analyzing qualitative data. All interviews and focus groups were recorded and transcribed verbatim. The interviews and focus groups transcripts were cleaned, deidentified and uploaded to Dedoose for qualitative coding, applying thematic analysis to identify patterns across the coded data regarding recruitment using the Consolidated Framework for Implementation Research (CFIR) [23].

#### Quantitative Study Design and Procedures

All five grantees submitted quantitative data on a quarterly basis, containing information about the practices, providers, and patients involved in the initiative. These data include the number and proportion of different types of participants (primary care practices, providers and staff, patients and community and other partners) recruited and retained by grantee projects, and the representativeness of those participants in the targeted population. The data from all five grantees used common metrics that were developed collaboratively across the five grantees and evaluation team in the first year of the project and were then aggregated and analyzed by a research programmer using SAS. For patient-level quantitative data, grantees reported, at the practice or (in one case) the grantee level, the total number of patients screened and the number of patients who screened positive for urinary incontinence. We calculated the percentage of patients screening positive by dividing the number of positives by the total number screened for each site, providing a standardized metric for comparing patient recruitment and detection rates across grantees.

We calculated simple descriptive statistics (means, sums, and percentages) for each grantee, as well as across grantees overall. If data were missing, we reached back to grantees to obtain the data or to confirm that the data were not collected.

## Results

Below we describe practice and provider recruitment strategies, perceptions of the success of the strategies, barriers to practice and provider recruitment, and metrics to describe the volume of recruitment and types of practices and providers that were recruited. We then describe grantees’ patient recruitment strategies, identify effective approaches to recruitment, and display metrics that quantify the volume of patients recruited.

### Practice and Provider Recruitment

All five grantees reported recruitment strategies that reflected there is no one-size-fits-all approach to recruiting practices and providers; strategies varied broadly across contexts. Grantees universally leveraged professional relationships, used practice facilitators, and conducted in-person and/or virtual meetings to engage with and recruit practices. Most also utilized email and/or Microsoft Teams to communicate with and invite practices to participate, often switching between channels to increase responsiveness. For instance, one grantee commented, “I’ll send an e-mail to certain clinics and certain providers, and they will not respond… then I have been using Microsoft Teams and then they respond instantly.” Leadership buy-in—such as support from organizational presidents, medical directors, and program chairs—was described as essential for fostering greater engagement, although some grantees reported difficulty obtaining leadership approval at large institutions. One grantee noted the challenges involved in obtaining leadership approval at large institutions.

We can’t go and talk to all the sites until we get approval from the medical director. Getting their [Medical Director(s)’] green lights was a little difficult. There was one medical director we met with who was not supportive of the project, so we could not approach her sites to implement this. When the medical director is behind the project, it can really help—especially if the medical director is actively advocating for the initiative at their site.

The layers of approval needed in large systems often made smaller, more “nimble” practices easier to recruit, though approval in large systems allowed for greater numbers of practices to be added at once. Additional strategies used by some grantees included webinars and educational events to provide information about MUI, and incentives for participation such as cash or continuing medical education (CME) credits. For instance, a grantee specifically offered, “…$3000 or $3500, but the practice doesn’t get that money until after the project is completed.” Another worked with a recruitment partner and sent out newsletters, noting, “Our recruitment partner did send our email to their 190 practices, and we [took] steps [in] coordinating with other recruitment partners.” Others leveraged social networks, including places of worship and social media postings, in efforts to reach providers, as captured by, “I posted in a Facebook group that is for physician moms, and I think we’ve enrolled three practices from my Facebook post.” Table 2 provides a summary of the strategies used to engage practices and providers.

**Table 2:** Provider and practice recruitment strategies and metrics, as of 2024

	All grantee data combined	Grantee A	Grantee B	Grantee C	Grantee D	Grantee E
**Practice and provider recruitment strategies as reported by qualitatively**						
Leveraging professional relationships	5/5	X	X	X	X	X
Practice facilitators	5/5	X	X	X	X	X
In-person meetings	5/5	X	X	X	X	X
Virtual meetings	4/5	X	X	X		X
Email/teams outreach	4/5		X	X	X	X
Webinars/Educational events	3/5		X	X		X
Leadership buy-in	3/5			X	X	X
Incentives	2/5		X			X
Social media	2/5		X			X
Print newsletters	1/5		X			
Recruitment partner	1/5		X			
**Practice recruitment metrics**						
Target number of participating practices	290	65	55	60	60	50
Number of practices invited to participate	666	74	343	79	110	60
Number of practices participating*	270	43	40	55	72	60
% Academic-affiliated	76%	100%	16%	69%	100%	
% Rural	9%	0%	41%	0%	6%	N/A
% Safety net	3%	0%	11%	4%	0%	
**Provider recruitment metrics**						
Number of primary Care providers (PCPs) per participating practice	8	10	7	11	4	5
Number of PCPs enrolled in the project*	1950	433	263	627	311	316
% Family medicine	30%	30%	N/A	16%	53%	N/A
% Internal medicine	53%	70%	N/A	45%	45%	N/A
% Geriatrics	3%	0%	N/A	7%	0%	N/A
% Ob/gyn	8%	0%	N/A	19%	0%	N/A
% Other PCP type	6%	0%	N/A	13%	2%	N/A

*Grantee E did not report characteristics of practices participating. Grantees B and E did not report provider counts by specialty

Recruiting practices was a widely shared challenge, particularly during early implementation. Barriers included provider burnout, gatekeeping by clinic leads, and layers of institutional approval. One grantee noted, “Many said they were significantly burned out.” Others described difficulty navigating clinic hierarchies: “There’s a gatekeeper feel… [as though we shouldn’t] engage directly [with providers].” Some teams adopted labor-intensive strategies, as illustrated by a principal investigator who described “driving far to meet one individual physician” to secure buy-in. Even when clinic champions were identified, staff turnover and limited availability could hinder momentum: “Sometimes I’ve had to change site champions because I just couldn’t get a hold of them.” The most consistent challenge was securing that first meeting—a key opportunity to frame the intervention as low-burden and worthwhile.

In quantitative analysis (Table 2), grantees who employed a combination of high-touch, relationship-driven strategies with supplementary digital communications (such as virtual meetings and email outreach) achieved the most successful recruitment outcomes. Grantees C, D, and E, all of whom reported strong leadership buy-in, met or surpassed their recruitment goals, with D and E exceeding targets by 20%. By contrast, grantee B employed the broadest set of strategies and invited the largest number of practices (343), but secured a lower conversion rate, particularly among rural clinics. This highlights the importance of relationship-building and leadership support as the most consistently effective approaches, while tactics such as webinars, incentives, and social media yielded more limited or variable impact. After 27 months of recruitment, three grantees had not yet reached their practice recruitment goals, while two surpassed theirs.

There was notable variation in recruited practice types: two grantees focused solely on academic-affiliated practices, while others achieved a mix of academic, rural, and safety-net clinics. The majority of providers enrolled were in geriatrics (54%) and family medicine (33%), with smaller percentages in obstetrics and gynecology, internal medicine, and other primary care areas. The number of providers per participating practice ranged from four to 12, averaging eight overall. Across all grantees, nearly 2000 primary care providers were recruited.

### Patient Recruitment

The grantees’ patient recruitment at the practice level, varied just as much as their practice recruitment (Table 3). Some grantees recruited patients through advertising in the waiting rooms, others reached out to patients via phone or letters in the mail. One grantee mentioned, “We had all of the surveys on clipboards ready to go and we would just say to the patients, ‘We’re conducting a survey… would you be able to fill this out?’”. Another grantee shared that,

“So at X clinic, we screen patients by telephone and actually there’s a higher likelihood of a patient screening because instead of possibly opening an email they’re always answering their phone and then once they’re on the phone they’re much more likely to participate.”

**Table 3:** Patient recruitment and screening strategies and metrics, as of 2024

	All grantee data combined	Grantee A	Grantee B	Grantee C	Grantee D	Grantee E
**Patient recruitment strategies**						
Questionnaire at check-in	4/5	X	X	X	X	
Pamphlets/advertising in waiting room	3/5			X	X	X
Bilingual outreach	3/5	X	X	X		
Newsletters/letters to patients	2/5				X	X
Educational videos	1/5		X			
**Patient screening strategies**						
Integrated into check-in workflow	3/5	X		X	X	
Phone screening	3/5		X	X		X
Screener on EHR	2/5	X	X			
**Patient recruitment metrics**						
Targeted number of patients to contact for screening/survey	45,550	25,000	2200	1350	10,000	7000
Number of patients who completed screening	134,852	111,806	605	3623	15,164	3654
Number of patients who screened positive for UI	28,978	14,944	146	1532	8819	3537

*CDS* clinical decision support (EHR-based); *APP* advanced practice professional

Three of the grantees specifically mentioned ensuring that their outreach materials were available in Spanish. One grantee emphasized, “We ensured that everything we handed out was available in English and Spanish.” Another grantee utilized educational videos as part of the recruitment process; another recruited as part of the consult process.

However, despite various recruitment efforts, the most effective approach was when practices integrated patient intake processes (e.g., Electronic Health Record (EHR) patient portal, registration desks, QR codes, etc.) into their workflow. Four grantees implemented a UI screener as part of the check-in process, allowing patients to complete it at home—integrated into the EHR by two grantees—or in the clinic prior to an appointment using paper or an electronic device (e.g., phone or tablet). One grantee, in their first year, stated, “We’re trying to get [our EHR] to have the screener in it so that the patients can complete the screening, and we will have some indicators for primary care physicians to encourage the patient to complete the screen there.” Integrating the screener into the EHR was aimed at streamlining the process, enabling providers to review screening results ahead of visits and better tailor care to the patient’s needs. This approach was reported as the most effective method for recruiting patients into the study to address UI. One grantee practice emphasized how seamless it was to recruit patients by incorporating screeners into their clinic intake process, noting that it was the most practical and effective approach. They stated, “I don’t know about other practices, but for us, it was pretty straightforward—like, ‘here’s how [EHR] will help us roll this out.’” Moreover, grantees that implemented integrated patient intake processes for recruitment and screening consistently exceeded their patient recruitment metrics, with two grantees nearly or more than doubling their target numbers (Table 3).

Comparing the patient recruitment and screening strategies across grantees reveals distinct patterns of effectiveness tied to their approach. All four grantees that integrated questionnaires into the check-in process (A, B, C, and D) demonstrated greater success in screening volumes, with those leveraging both bilingual outreach and embedded workflow screening (A, B, and C) particularly successful relative to their initial targets. For example, grantee A, which combined questionnaire intake, bilingual outreach, educational videos, and EHR-based screeners, vastly exceeded its recruitment goal—screening over 111,000 patients compared to a target of 25,000—and identified nearly 15,000 women positive for UI. Grantee B, despite deploying several strategies including bilingual outreach and educational videos, screened a modest number relative to its target (605 out of 2200), suggesting that workflow integration alone may not be sufficient without concurrent broad outreach or engagement. Grantee C, using check-in questionnaires, pamphlet advertising, and bilingual outreach, more than doubled its screening target and achieved high detection rates. Grantees D and E, both of which relied more heavily on patient-facing materials in waiting rooms and newsletters rather than bilingual outreach, achieved more moderate yet substantial numbers, with D screening over 15,000 patients (against a 10,000 target) and E screening over 3600 (against a 7000 target).

Regarding screening strategies, those who embedded the screening into the patient check-in process or EHR workflow screened substantially higher numbers of patients compared to those relying predominantly on outreach materials alone. Use of phone screening (by B, C, and E) helped engage some patients, but appeared less scalable than integrated workflow approaches. Across all grantees, implementation of bilingual resources helped ensure equitable reach, but the highest numbers were screened where multiple, complementary strategies—particularly workflow integration—were used. Overall, workflow-integrated and bilingual strategies were consistently associated with exceeding or meeting patient recruitment and screening goals, while less integrated approaches or those relying on passive outreach yielded lower patient engagement.

We also found that practices with staff who were engaged and invested in the initiative were more likely to be successful at recruiting patients. As one grantee explained,

“There are some [practices] that are really owning the resources, versus others [that are] just using the resources. They’re making [the resources] work, they work well for them. They’re handing them out to all their patients. Versus, some might have forgotten that those resources exist, or they might have lost that email and might not really be using them at all.”

These various strategies coincided with three (grantees A, C, and D) achieving their patient recruitment metrics (Table 3). Across the grantees, 105,470 patients were screened for UI and 23% (24,572 women), screened positive for UI.

## Discussion

This study evaluated the comparative effectiveness of recruitment strategies for practices, providers, and patients within a dissemination and implementation initiative for evidence-based nonsurgical treatments for urinary incontinence (UI) in women. Our findings highlight several themes that are best understood through the lens of the CFIR.

Within the *process* domain, practice facilitation and structured meetings emerged as consistently used strategies across grantees, facilitating engagement at both the provider and practice levels. These interpersonal approaches helped navigate logistical challenges and built trust—a finding echoed in implementation science literature, which emphasizes the importance of relationship-building and tailored engagement [24, 25]. While digital outreach and incentives complemented these efforts, they were not fundamental drivers of recruitment success. The limited impact of financial incentives aligns with evidence suggesting that incentive effectiveness depends on context, perceived value, and alignment with intrinsic motivations [26].

The *inner setting* domain—particularly organizational culture and readiness for implementation—played a pivotal role. Grantees that secured institutional leadership buy-in tended to meet or exceed recruitment targets, suggesting that leadership endorsement facilitated resource allocation and staff engagement. However, it is important to note that our data are qualitative; we observed a pattern but did not conduct statistical tests to establish a causal relationship. This interpretation is supported by prior research showing leadership support as an enabler of adoption and implementation [27, 28]. Nevertheless, without quantitative association analyses, our findings should be considered as indicative and context-dependent rather than definitive.

For *patient recruitment*, integrating UI screening into intake workflows—leveraging the *intervention characteristics and process* domains—proved highly effective. Practices that embedded screening into check-in or EHR workflows reached greater numbers of eligible patients, consistent with evidence supporting the use of “opt-out” and workflow-integrated approaches to promote recruitment and intervention uptake [29, 30]. Furthermore, practices with engaged staff fostered a collaborative environment that enhanced patient participation, underscoring the importance of the *characteristics of individuals* domain in implementation. Engagement of practice champions and staff investment not only increased recruitment but also improved follow-up and care quality, echoing prior studies on the role of committed personnel in successful implementation [31–33].

Grantee B’s experience illustrates challenges that arise when scaling recruitment across diverse settings. Despite employing the most recruitment strategies and targeting a mix of academic, rural, and safety-net sites, structural and contextual barriers—such as geographic dispersion and variable resources—impeded recruitment. This finding supports CFIR’s emphasis on the importance of *outer setting* and *structural characteristics* in shaping implementation outcomes.

Although our study did not collect formal cost or resource utilization data, several qualitative insights gathered during implementation highlight important considerations for decision-makers regarding workload and staffing. Embedding screening and recruitment processes into the EHR workflow, while effective for patient identification and engagement, required significant up-front investment of staff time for training, workflow redesign, and ongoing EHR support. Additionally, sites that achieved higher recruitment numbers often described added administrative and clinical responsibilities for existing personnel—such as managing survey distribution, following up with positive screens, and documenting in the EHR—which sometimes resulted in increased workload and the need for role adjustments. Practices with engaged, well-coordinated teams were able to more seamlessly absorb these responsibilities, while others reported strain, particularly in settings with limited staffing or competing priorities. These findings suggest that, when planning future large-scale screening initiatives, stakeholders should consider not only technical and financial resources but also the human resource requirements and potential impact on staff workload and workflow. Incorporating dedicated support, streamlining EHR integration, and providing ongoing training may help address these barriers and facilitate sustainable implementation.

### Limitations

This analysis has four main limitations. First, we are comparing five grantees that differed in many respects, and it is therefore not possible to identify which aspects of the recruitment process were ultimately responsible for recruitment outcomes. However, the qualitative data and perspectives of the staff involved in the initiatives provide insights into possible influences. Second, there was variation in the types of quantitative data that the grantees provided. Some grantees initiatives did not yield the same types of data, and some grantees had privacy policies that did not allow reporting of demographic data at the individual patient, provider, or practice level. These discrepancies are mostly explained by the history of the project: each grantee was initially funded to study a unique intervention, and after they had designed their interventions, AHRQ requested that each grantee collect common metrics data so that the projects could be studied in the aggregate. However, owing to the differences across projects, it was not always possible for grantees to conform their data to fit the common metric definition. For example, grantees collected patient-level data on different population sub-groups: one grantee collected patient-level data for everyone who screened positive, another reported patient-level data only for those who completed a follow-up visit, and another reported demographic data for everyone who completed a survey, regardless of UI screening outcome. As a result, it was not possible to compare patient-level demographic data across grantees or to fully understand the characteristics of individuals who screened positive for UI. Third, the grantees implemented their initiatives within very different populations and systems of care, making comparisons across grantees difficult. However, this heterogeneity also allows generalization to more diverse primary care practices, as not every practice was located in an academic setting and the practices are geographically located in different regions of the country. Finally, our analysis plan did not allow for qualitative data collection from patients, resulting in a lack of patient perspectives on the acceptability of recruitment and screening processes. This absence of patient voices limits our ability to draw conclusions about the true acceptability of these strategies and their potential impact on UI-related stigma reduction.

## Conclusion

Our study suggests that the most successful recruitment strategies were those that leveraged interpersonal relationships and embedded processes within existing workflows, particularly when supported by organizational leadership and staff engagement. The relative ineffectiveness of standalone incentives and passive outreach underscores the need for multi-component, contextually tailored strategies. Our interpretations are predominantly qualitative; future research with multivariable quantitative analyses will be necessary to clarify predictors of recruitment success and further elucidate the mechanisms underlying effective implementation. Mapping findings to CFIR enriches our understanding and provides a robust framework for future intervention design and evaluation.

## Data Availability

Data is available with approval from the Agency of Healthcare Research and Quality.
